# Association of Alanine Aminotransferase With Different Metabolic Phenotypes of Obesity in Children and Adolescents: The CASPIAN-V Study

**DOI:** 10.3389/fendo.2020.00358

**Published:** 2020-08-07

**Authors:** Roya Kelishadi, Zeinab Hemati, Mostafa Qorbani, Mohammad Esmaeil Motlagh, Shirin Djalalinia, Zeinab Ahadi, Gita Shafiee, Armita Mahdavi Gorabi, Hadith Rastad, Hasan Ziaodini, Seyede Shahrbanoo Daniali, Ramin Heshmat

**Affiliations:** ^1^Pediatrics Department, Child Growth and Development Research Center, Research Institute for Primordial Prevention of Non-communicable Disease, Isfahan University of Medical Sciences, Isfahan, Iran; ^2^Non-communicable Diseases Research Center, Alborz University of Medical Sciences, Karaj, Iran; ^3^Endocrinology and Metabolism Research Center, Endocrinology and Metabolism Clinical Sciences Institute, Tehran University of Medical Sciences, Tehran, Iran; ^4^Pediatrics Department, Ahvaz Jundishapur University of Medical Sciences, Ahvaz, Iran; ^5^Development of Research and Technology Center, Deputy of Research and Technology, Ministry of Health and Medical Education, Tehran, Iran; ^6^Chronic Diseases Research Center, Endocrinology and Metabolism Population Sciences Institute, Tehran University of Medical Sciences, Tehran, Iran; ^7^Social Determinants of Health Research Center, Alborz University of Medical Sciences, Karaj, Iran; ^8^Bureau of Health and Fitness, Ministry of Education and Training, Tehran, Iran

**Keywords:** alanine aminotransferase, obesity, metabolic syndrome, children and adolescents, Iran

## Abstract

**Aim:** To determine the association of alanine aminotransferase with different metabolic phenotypes of obesity in a nationally- representative sample of Iranian children and adolescents.

**Methods:** This national study was conducted in the framework of the fifth survey of a national surveillance program entitled Childhood and Adolescence Surveillance and Prevention of Adult Non-communicable Disease study. Participants consisted of 4,200 subjects aged 7–18 years, who were recruited by multistage random cluster sampling from 30 provinces in Iran. They were categorized to normal weight and obese groups and in each group those with and without MetS components.

**Results:** Overall, 3,843 of participants completed the survey (response rate: 91.5%). Their mean (SD) age was 12.58 (3.15) years; 52.6% were boys, and 72.7% lived in urban areas. Mean of alanine aminotransferase (ALT) in subjects with abdominal obesity and general obesity was 8.81 (95% CI: 7.99–9.62) (IU/L) and 8.87 (95% CI: 7.28–10.46) (IU/L), respectively. According to the adjusted model, one unit increment in ALT increased odds of being metabolically non-healthy obese (MNHO) by 2% compared to metabolically healthy non-obese (MHNO) [adj.OR (95% CI): 1.02 (1.01–1.04)]. Also, subjects in the third and fourth quartiles of serum ALT had significantly greater odds of being MNHO than those in its first quartile [Q3/Q1: adj. OR (95% CI): 3.85 (1.70–8.71); Q4/Q1: Adj. OR (95% CI): 3.63 (1.51–8.73)].

**Conclusion:** This large population-based study revealed significant associations between metabolic phenotypes of obesity and ALT level.

## Introduction

Childhood obesity and metabolic syndrome (MetS) has become a worldwide problem and is no longer only limited to high-income countries (1, 2).

Serum alanine aminotransferase (ALT) is the liver enzyme most strongly correlated with liver fat accumulation (3), a surrogate marker for non-alcoholic fatty liver disease (NAFLD) (4). Besides, obese children with modest elevations of ALT might present metabolic impairment including deterioration in insulin sensitivity and glucose tolerance, hypertriglyceridemia, and hypoadiponectinemia (5).

While a large number of studies showed the association of liver enzymes with cardio-metabolic components and metabolic syndrome (6, 7), a few studies have examined the association of these enzymes with the different metabolic phenotypes of obesity, especially in children and adolescents (8–10). Therefore, this study aims to determine the association of ALT level with different metabolic phenotypes of obesity in children and adolescents.

## Methods

The present study was a part of “the fifth survey of the school-based surveillance system entitled **C**hildhood and **A**dolescence **S**urveillance and **P**revent**i**on of **A**dult **N**on-communicable Disease (CASPIAN-V) study” (2014-2015) conducted in 30 provinces of Iran. Details on the study protocol have been discussed previously (11), and we briefly point to the main parts.

### Study Population and Sampling

Using the multistage stratified cluster sampling method, the study participants were selected from 7 to 18-year-old school subjects living in urban and rural areas of 30 provinces.

In each province, proportional to the size sampling method which was adopted to obtain a representative sample of students from rural/urban area and from primary/secondary school grades, with an equal sex ratio (12).

Achieving the desired number of samples was obtained using cluster sampling in each province with equal cluster sizes. Clusters were determined at school levels. The size of each cluster was 10 subjects; meaning that a total of 10 statistical units (including 10 subjects and their parents) would be considered in each cluster. The sample size of the main survey included 480 subjects in each province (48 clusters of 10 subjects), i.e., a total of 14,400 subjects at the national level. In each province, 14 out of 48 clusters were randomly selected for biochemical tests. Therefore, the sample size of the current study was estimated to be 4,200.

### Procedure and Measurements of Data Gathering

#### Demographic Information

Through an interview with parents or child, demographic information was asked for all subjects in the sampled classes of the selected schools. Family-based characteristics including family history of chronic diseases (hypertension, dyslipidemia, diabetes, and obesity), parental level of education (the highest total years of schooling), possessing a family private car and the type of home (rented/owned), dietary behaviors, physical activity (PA), and sedentary lifestyle were all considered.

### Questionnaires

Data for subjects gathered through the Persian-translated version of the questionnaire was developed based on the World Health Organization-Global School Student Health Survey (WHO-GSHS) (13). The validity and reliability of questionnaires have been assessed through previous assessments (14).

Moreover, demographic information including age, sex, family history of diseases and metabolic risk factors, complementary data on family characteristics, namely household size, the birth order of subjects, and socioeconomic variables were questioned through parents' questionnaires.

#### Measurements

Under standard protocols and by using calibrated instruments, a team of trained health care experts performed the physical examination. Weight was measured in light clothing to the nearest 0.1 kg on a SECA digital weighing scale (SECA, Germany). Height was assessed without shoes to the nearest 0.1 cm while the subjects were standing, and the shoulders were in normal position (13).

We calculated Body mass index (BMI) by dividing weight (kg) to height squared (*m*^2^) and categorized it using the WHO growth charts (13).

We measured waist circumference (WC) using a non-elastic tape at a point midway between the lower border of the rib cage and the iliac crest at the end of normal expiration to the nearest 0.1 cm. We also measured Hip circumference at the widest part of the hip at the level of the greater trochanter to the nearest 0.1 cm.

Blood pressure was measured in the sitting position on the right arm using a mercury sphygmomanometer with an appropriate cuff size. It was measured two times at 5-min intervals; systolic and diastolic pressures were recorded and the average was registered (15).

Physical activity (PA): Through a validated questionnaire, information of weekly frequency of leisure time PA outside the school was collected, for the previous week (14). In this regard; at least 30 min duration of exercises per day, that caused heavy sweating or large increases in breathing or heart rate, was defined as PA. The response options were categorized as; none, 1–2 days, 3–6 days, and every day. For statistical analysis, each weekly frequency categorized into two groups; 0–3 days per week (Low); 4–7 days (High) (16).

Screen time (ST): The ST behavior of the children was assessed through the questionnaire that asked them to report the average number of hours/days they spent on watching Television (TV)/Video compact discs (VCDs), personal computer (PC), or electronic games (EG) in time of weekdays and weekends.

Socioeconomic status (SES): For assessment of the subjects' SES, we used principal component analysis (PCA) method by including questions related to parental education, parents' job, possessing a private car, school type (public/private), and having a personal computer which were combined as a unique index (11).

#### Laboratory Analysis

Selected subjects for blood sampling were referred to the predefined laboratory. After a 12-h overnight fasting 6 mL venous blood sample was collected. All collection tubes were centrifuged at 2500–3000 × g for 10 min. Immediately after centrifugation, serum samples were aliquot into 200 microliter tubes and stored at −70° C. Using a comprehensive by cold chain program, all of the samples were transferred to Isfahan Mahdieh Laboratory. Alanine aminotransferase (ALT), Fasting blood glucose (FBG), triglycerides (TG), total cholesterol (TC), low- density lipoprotein-cholesterol (LDL-C), and high-density lipoprotein-cholesterol (HDL-C) were measured enzymatically by Hitachi auto-analyzer (Tokyo, Japan) (17, 18).

#### Definitions

##### Metabolic syndrome (MetS) and its indicators

We used the Adult Treatment Panel III (ATP III) criteria modified for children and adolescents; participants were considered as having MetS if they met at least three of the five cardiometabolic risk factors: abdominal obesity, elevated BP, elevated FBG, high serum TG, and low serum HDL (19, 20). We defined these five cardiometabolic risk factors as below:

Abdominal obesity: waist-to-height ratio ≥ 0.5; elevated BP: BP ≥ 90^th^ percentile, elevated FBG: FBG ≥ 100 mg/dL, high serum TG: Serum TG ≥ 100 mg/dL; and low serum HDL: HDL-C ≤ 40 mg/dl.

##### Obesity

We used the WHO growth curves to define BMI categories, (13), i.e., age and sex-specific BMI in the 85th to 95th percentile was considered overweight, and age and sex-specific BMI ≥ 95th percentile as obesity.

Also, according to the BMI status, our participants fall into four different phenotypes of obesity groups:

- Normal: 5th <BMI<85th percentile and waist-to-height ratio (WHtR) < 0.5,
- Only abdominal obesity: WHtR>0.5 and BMI<95th percentile,
- Only general obesity: BMI>95th and WHtR<0.5,
- Combined obesity: BMI>95th and WHtR>0.5.

##### Metabolic phenotypes of obesity

Participants were classified into four groups according to their obesity and metabolic syndrome status:

1. Metabolically Healthy Obese (MHO): Obese individuals without metabolic syndrome.
2. Metabolically non-healthy Non-Obese (MNHNO): Non-obese individuals with metabolic syndrome.
3. Metabolically Non-healthy Obese (MNHO): Obese individuals with metabolic syndrome.
4. Metabolically Healthy Non-Obese (MHNO): Non-obese individuals without metabolic syndrome.

### Ethical Concerns

The Research and Ethics council of Isfahan University of Medical Sciences reviewed and approved the Study protocol (Project number: 194049). After a complete explanation of the study objectives and protocols, written informed consent was obtained from the parents of participants older than 16 years old in this study, while oral consent was obtained from the participants under 16 years.

### Statistical Analysis

Using STATA package ver. 11.0 (Stata Statistical Software: Release 11. College Station, TX: Stata Corp LP. Package). All statistical measures were estimated using survey data analysis methods. We summarized continuous variables by mean and standard deviation (SD), and categorical variables by number (percentage).

Mean of ALT between different phenotypes of obesity and metabolic syndrome was compared by analysis of variance (ANOVA) and Tukey *post hoc* test.

The association of ALT with different (metabolic) phenotypes of obesity was assessed using crude and adjusted multinominal regression models. To perform regression analysis, serum ALT levels were also divided into four quartiles (Q) (Q1: ≤ 5 IU/L [*n* = 1,093], Q2: 6-7 IU/L [*n* = 983], Q3: 8-10 IU/L [*n* = 968], Q4: ≥ 11 IU/L [*n* = 799]). We reported crude and adjusted ORs for the second, third, and fourth quartiles of ALT levels compared to its first quartile.

In the adjusted model, the association was adjusted for age, gender, ST, PA, SES, and living area. Results of multinomial logistic regression presented as odds ratio (OR) and 95% confidence interval (CI). *P*-value of < 0.05 was considered as statistically significant.

## Results

From the 4,200 invited subjects, 3,843 participants completed the survey (response rate: 91.5%). Their mean (SD) age was 12.58 (3.15) years; 52.6% were boys, and 72.7% lived in urban areas.

The mean of ALT in subjects with abdominal obesity and general obesity was 8.81 (95% CI: 7.99–9.62) (IU/L) and 8.87 (95% CI: 7.28–10.46) (IU/L), respectively. As presented in Table 1, the mean level of ALT had no statistical difference across various phenotypes of obesity (*P* = 0.11).

**Table 1:** Mean (95% CI) of Alanine aminotransferase (ALT) according to the different phenotypes of obesity regardless of MetS status: the CASPIAN-V study.

**Groups**	**ALT (IU/L)**		
	**Mean**	**95% CI**	***P*-value**
Normal^a^	8.19	7.95–8.45	0.11
Only abdominal obesity^b^	8.81	7.99–9.62	
Only general obesity^c^	8.87	7.28–10.46	
Combined obesity^d^	8.94	8.29–9.59	

a *Normal: 5th < BMI <85^th^ percentile and WHtR <0.5*.

b *Only Abdominal obesity: WHtR > 0.5 and BMI < 95^th^ percentile*.

c *Only Genalarized obesity: BMI > 95^th^ and WHtR < 0.5*.

d *Combined Obesity: BMI > 95^th^ and WHtR > 0.5*.

*P ≤ 0.05 is considered as significant.

*ALT, Alanine aminotransferase; CI, confidence interval*.

Table 2 shows that the mean level of ALT was significantly different across the metabolic phenotypes of obesity (*P* =0.002). Results of the *post hoc* test showed that the mean level of ALT was higher in the MNHO group compared to the MHNO, MHO, and MNHNO groups (*P* < 0.05).

**Table 2:** Mean (95% CI) of Alanine aminotransferase (ALT) according to the different metabolic phenotypes of obesity: the CASPIAN-V study.

**Groups**	**ALT (IU/L)**			
	**Mean**	**95% CI**	***P*-value between groups**	***P*-value within groups**
MHNO^a^	8.27	8.02–8.52	0.002	0.98^a^^b^
MHO^b^	8.41	7.81–9.01		0.90^a^^c^
MNHNO^c^	8.72	7.70–9.74		0.001^a^^d^
MNHO^d^	11.58	9.33–13.84		0.97^b^^c^
				0.005^b^^d^
				0.042^c^^d^

a *MHNO, Metabolic Healthy Non Obese (healthy)*.

b *MHO, Metabolic Healthy Obese*.

c *MNHNO, Metabolic Non Healthy Non Obese*.

d *MNHO, Metabolic Non Healthy Obese*.

*P ≤ 0.05 is considered as significant.

*ALT, Alanine aminotransferase, CI, confidence interval*.

The association of ALT concentration with different obesity phenotypes in multinominal regression models is presented in Table 3. Overall, it shows no significant association between the ALT concentration and the different phenotypes of obesity (general and abdominal obesity) in crude and adjusted models.

**Table 3:** Association of Alanine aminotransferase (ALT) with different phenotypes of obesity regardless of the MetS status in multinomial logistic regression models: the CASPIAN- V study.

**Group /variable**		**Only abdominal obesity^a^**		**Only general obesity^b^**		**Combined Obesity^c^**	
		**Model I^d^**	**Model II^e^**	**Model I^d^**	**Model II^e^**	**Model I^d^**	**Model II^e^**
		**Crude OR (95% CI)**	**Adjusted OR (95% CI)**	**Crude OR (95% CI)**	**Adjusted OR (95% CI)**	**Crude OR (95% CI)**	**Adjusted OR (95% CI)**
ALT (Quartiles)	Q2/Q1	0.97 (0.74–1.29)	1.03 (0.76–1.39)	1.00 (0.59–1.69)	1.03 (0.58–1.80)	1.01 (0.72–1.41)	1.05 (0.73–1.51)
	Q3/Q1	1.19 (0.91–1.56)	1.24 (0.93–1.66)	0.71 (0.39–1.26)	0.75 (0.41–1.39)	1.26 (0.91–1.74)	1.21 (0.85–1.72)
	Q4/Q1	1.09 (0.82–1.46)	1.15 (0.85–1.57)	1.05 (0.60–1.81)	1.02 (0.56–1.86)	1.41 (1.01–1.96)*	1.28 (0.89–1.83)
ALT (IU/L)		1.01 (0.99–1.02)	1.00 (0.99–1.02)	1.01 (0.99–1.03)	1.01 (0.99–1.03)	1.01 (0.99–1.02)	1.01 (0.99–1.02)

a *Only Abdominal obesity: WHtR>0.5 and BMI <95th percentile*.

b *Only Genalarized obesity: BMI>95th and WHtR <0.5*.

c *Combined Obesity: BMI>95th and WHtR>0.5*.

d *Crude model*.

* *p < 0.05*.

e *Adjusted for age, gender, physical activity, screen time, socioeconomic status, and living area*.

*ALT, Alanine aminotransferase; OR, odds ratio CI: confidence interval*.

*ALT Quartiles: Q1: ≤ 5 IU/L [n = 1,093], Q2: 6-7 IU/L [n = 983], Q3: 8-10 IU/L [n = 968], Q4: ≥ 11 IU/L [n = 799]*.

Table 4 presents the association of ALT with different metabolic phenotypes of obesity in multinomial regression models. According to the adjusted model, one unit increment in ALT increased odds of being MNHO (compared to MHNO**)** by 2% [Adj. OR (95% CI): 1.02 (1.01–1.04)].

**Table 4:** Association of Alanine aminotransferase (ALT) with different metabolic phenotypes of obesity in multinomial logistic regression models: the CASPIAN- V study.

**Group /variable**		**MHO^a^**		**MNHNO^b^**		**MNHO^c^**	
		**Model I^d^**	**Model II^e^**	**Model I^d^**	**Model II^e^**	**Model I^d^**	**Model II^e^**
		**Crude OR (95% CI)**	**Adjusted OR (95% CI)**	**Crude OR (95% CI)**	**Adjusted OR (95% CI)**	**Crude OR (95% CI)**	**Adjusted OR (95% CI)**
ALT (Quartiles)	Q2/Q1	0.94 (0.69–1.28)	0.95 (0.68–1.33)	1.35 (0.80–2.27)	1.58 (0.90–2.77)	2.28 (0.97–5.36)	2.45 (0.99–6.05)
	Q3/Q1	0.96 (0.71–1.31)	0.90 (0.65–1.27)	1.50 (0.90–2.50)	1.64 (0.93–2.87)	3.18 (1.41–7.19)*	3.85 (1.70–8.71)*
	Q4/Q1	1.09 (0.79–1.49)	1.00 (0.71–1.41)	1.35 (0.78–2.33)	1.47 (0.81–2.67)	3.24 (1.35–7.73)*	3.63 (1.51–8.73)*
ALT (IU/L)		1.00 (0.98–1.02)	1.00 (0.98–1.00)	1.01 (0.98–1.02)	1.00 (0.98–1.03)	1.01 (1.00–1.03)*	1.02 (1.01–1.04)*

a *MHO, Metabolic Healthy Obese*.

b *MNHNO, Metabolic Non Healthy Non Obese*.

c *MNHO, Metabolic Non Healthy Obese*.

d *Crude model*.

e *Adjusted for age, gender, physical activity, screen time, socioeconomic status, and living area*.

* *P < 0.05*.

*ALT, Alanine aminotransferase; CI, confidence interval; OR, odds ratio*.

*ALT Quartiles: Q1: ≤ 5 IU/L [n = 1,093], Q2: 6-7 IU/L [n = 983], Q3: 8–10 IU/L [n = 968], Q4: ≥ 11 IU/L [n = 799]*.

Adjusted multinomial regression analysis also demonstrated that subjects in the third and fourth quartiles of ALT level compared to those in its first quartile were more likely to be MNHO [Q3/Q1: Adj.OR (95% CI): 3.85 (1.70–8.71); Q4/Q1: Adj.OR (95% CI): 3.63 (1.51–8.73)].

## Discussion

We found that the MNHO group has a higher mean ALT level compared to MHNO and MHO groups. According to the results from adjusted multinominal regression models, one unit increment in ALT increased odds of being MNHO (compared to MHNO) by 2% independent of age, gender, physical activity, screen time, socioeconomic status, and living area. However, we did not detect any significant association between ALT level with different forms of obesity (only abdominal obesity, only general obesity, and combined obesity) regardless of the MetS status.

In line with our study, Aldhoon-Hainerová et al. found that alanine aminotransferase could differentiate MNHO from Metabolically Health without abdominal obesity in both sexes in adolescence and from Metabolically Health regardless of waist circumference obesity in boys (8). Likewise, Suárez-Ortegón et al. observed a higher serum ALT level in children with MNHO (vs. MHO), and metabolically unhealthy overweight (vs. metabolically healthy overweight) (9). Xie et al. in a cross-sectional study on 2,197 obese (BMI ≥25 kg/m2) adults older than 40 years observed that the ALT might be an effective marker for identifying MNHO in this population (10).

Besides, in a study in children and adolescents, those with hypertriglyceridemic-waist phenotype had a higher level of ALT than those without this phenotype (21).

Individuals with MNHO have an increased level of liver enzymes and are at increased risk for hepatic steatosis (22, 23).

Higher levels of liver enzymes, especially ALT, may indicate higher liver fat deposition. Liver fat is a useful tool to assess metabolic health status (24).

We did not find significant differences between MHO and MNHNO with MHNO regarding the ALT serum level. Some other studies also documented that MHO individuals have lower levels of ectopic liver fat and potentially lower risk of NAFLD (25, 26).

In contrast, a study reported that the prevalence of NAFLD was similar in MHO and overweight participants, but higher than those with normal weight (26). On the other hand, the concept of MNHNO is of concern both in adults and in pediatric populations (27, 28). However, some obese individuals have no metabolic impairment it should be considered that the MHO individuals are not healthy, and in spite of their normal metabolic profile, they might face other adverse health effects of obesity (29). These individuals are a high-risk group for insulin resistance, cardiovascular risk diseases, and related mortality (30, 31).

We did not detect any significant association between ALT level and different phenotypes of obesity regardless of MetS status (general/abdominal obesity). In contrast with our findings, some previous studies have documented the association of ALT with abdominal obesity (32, 33). In overweight children and adolescents, for each 5- cm increase in WC and every 1-point increase in BMI z- score, there was a 1.3-fold higher risk of having increased ALT levels (34). Compared with general obesity, abdominal obesity is more strongly correlated with metabolic impairment (35). Besides, a study using data from an ultrasonography survey revealed that trunk fat was associated with increased serum ALT levels independently of anthropometric indices of general and abdominal obesity (36). Also, in children diagnosed with NAFLD, the strongest correlates to increased serum ALT levels across the spectrum of body weights were the abdominal skinfolds and the sum of the trunk skinfolds (37). In another study, multiple linear regression adjusted for several covariates found that obesity assessed by abdominal height was a better correlate of ALT levels than BMI (38).

### Limitations and Strengths

The main limitation of this study is the cross-sectional nature of the findings, which infer the causality of the associations. Moreover, we could not examine the pubertal status, and some of our findings might be influenced by puberty. The strengths of the study are the novelty in the pediatric population, the large sample size, and considering various phenotypes of obesity.

## Conclusion

This large population-based study provides information on the association of ALT levels with different metabolic phenotypes of obesity in children and adolescents. MNHO group may have a higher mean ALT level compared to MHNO and MHO groups. Our findings although suggest that ALT level has not association with different phenotypes of obesity regardless of the MetS status (only abdominal obesity, only general obesity, and combined obesity).

## Data Availability Statement

The data are obtained from a national surveillance program, so they cannot be accessed publicly; they can be provided after the approval of the steering committee of the project. Requests to access the datasets should be directed to mqorbani1379@yahoo.com.

## Author Contributions

RK and MQ participated in the sequence alignment and drafted the article. ZH participated in the study design, final revision, and edition. HR, GS, and RH participated in the sequence alignment and drafted the manuscript. MM and SD participated in the study design and interpretation. HZ and ZA participated in the data acquisition. MQ, SSD, and AM participated in the design of the study and performed statistical analysis. All authors read and approved the final manuscript.

## Conflict of Interest

The authors declare that the research was conducted in the absence of any commercial or financial relationships that could be construed as a potential conflict of interest.
